# Modelling the Stiffness-Temperature Dependence of Resin-Rubber Blends Cured by High-Energy Electron Beam Radiation Using Global Search Genetic Algorithm

**DOI:** 10.3390/polym12112652

**Published:** 2020-11-11

**Authors:** Ivan Kopal, Juliána Vršková, Alžbeta Bakošová, Marta Harničárová, Ivan Labaj, Darina Ondrušová, Jan Valíček, Jan Krmela

**Affiliations:** 1 Department of Numerical Methods and Computational Modeling, Faculty of Industrial Technologies in Púchov, Alexander Dubček University of Trenčín, Ivana Krasku 491/30, 020 01 Púchov, Slovakia; ivan.kopal@tnuni.sk (I.K.); alzbeta.bakosova@student.tnuni.sk (A.B.); ivan.labaj@tnuni.sk (I.L.); krmela@tnuni.sk (J.K.); 2 Department of Material Technologies and Environment, Faculty of Industrial Technologies in Púchov, Alexander Dubček University of Trenčín, Ivana Krasku 491/30, 020 01 Púchov, Slovakia; juliana.vrskova@student.tnuni.sk (J.V.); darina.ondrusova@tnuni.sk (D.O.); 3 Department of Electrical Engineering, Automation and Informatics, Faculty of Engineering, Slovak University of Agriculture in Nitra, Tr. A. Hlinku 2, 949 76 Nitra, Slovakia; jan.valicek@uniag.sk; 4 Department of Mechanical Engineering, Institute of Technology and Business, Faculty of Technology, České Budějovice, Okružní 10, 370 01 České Budějovice, Czech Republic; 5 Department of Transport Means and Diagnostics, Faculty of Transport Engineering, University of Pardubice, Studentská 95, 532 10 Pardubice, Czech Republic

**Keywords:** resin-rubber blends, dynamic mechanical analysis, Weibull distribution, genetic algorithm, electron-beam irradiation

## Abstract

Modelling the influence of high-energy ionising radiation on the properties of materials with polymeric matrix using advanced artificial intelligence tools plays an important role in the research and development of new materials for various industrial applications. It also applies to effective modification of existing materials based on polymer matrices to achieve the desired properties. In the presented work, the effects of high-energy electron beam radiation with various doses on the dynamic mechanical properties of melamine resin, phenol-formaldehyde resin, and nitrile rubber blend have been studied over a wide temperature range. A new stiffness-temperature model based on Weibull statistics of the secondary bonds breaking during the relaxation transitions has been developed to quantitatively describe changes in the storage modulus with temperature and applied radiation dose until the onset of the temperature of the additional, thermally-induced polymerisation reactions. A global search real-coded genetic algorithm has been successfully applied to optimise the parameters of the developed model by minimising the sum-squared error. An excellent agreement between the modelled and experimental data has been found.

## 1. Introduction

Highly cross-linked resins and their blends that are susceptible to brittle failure can be effectively toughened by blending them with various elastomers [1]. A resole type of phenol-formaldehyde resin in combination with melamine resin results in a blend with universal properties that predispose it to broader use in the various types of industry [2]. The most commonly used and commercially available elastomeric material for improving the performance of resin blends is nitrile rubber, which allows targeted modification of their deformation behaviour and helps to improve mechanical properties determined primarily by volume fraction and strength of individual phases of the blend, the degree of phase separation and adhesion between them [3]. A blend of melamine resin and phenol-formaldehyde resin with nitrile rubber—which combines the thermal and dimensional stability and easy of moulding of phenolics with the wide range of colourability of melamine resins and the toughness as well as the flexibility of elastomers—is commonly used as a polymeric matrix for various composite systems in a number of practical applications, particularly in the automotive industry [4]. However, the curing of such resin-rubber blends is a time-consuming and energy-intensive process that significantly burdens the environment. The curing of the resin-rubber blends by high-energy ionising radiation, such as electron beam (EB) and gamma rays radiation, as well as the modification of polymers sensitive to radiation in order to achieve the desired properties in general, in many practical applications, can be an efficient, relatively inexpensive and more environmentally friendly alternative to traditional thermal curing [5].

Generally, on exposure to high-energy ionising radiation, the macromolecules of irradiated polymeric materials become excited or ionised after absorption of energy of incident radiation (particles or photons). The excited macromolecules are able to initiate a number of chemical processes (such as fragmentation to carbocations and free radicals, the capture of electrons by polymeric and oxygen molecules, dissociative capture of electrons, and others [6]), producing highly reactive forms (such as free neutral radicals, radical anions and cations, low-energy electrons, singled and triplet state of excited macromolecules, and others), which usually leads to simultaneous cross-linking and degradation reactions [7]. The radiation-induced cross-linking occurs mostly between two adjacent macromolecular chains due to breakage of their side chains. The free chain radicals finally react with each other leading to the formation of a chemical cross-link accompanied by an increase in molecular weight. At lower radiation doses, cross-linking has a lateral character, while irradiation with higher radiation doses leads to the formation of a three-dimensional polymeric network [8]. On the other hand, radiation-induced degradation of irradiated polymers occurs by breakage of the main chains of the polymeric material, leading mainly to a decrease in its molecular weight [9]. During irradiation, both these phenomena coexist, while the predominance of one of them over the other depends on several factors, such as the initial molecular structure and morphology of the polymeric material, type of radiation, and irradiation conditions, including the size of the applied radiation dose, and many others [10].

Modelling the effect of radiation on the resulting properties of irradiated polymer plays an important role in the research and development of new materials for various industrial applications, as well as the effective modification of existing materials based on polymer matrices in order to achieve the required properties. The aim of the presented work is to model the influence of high-energy EB radiation on the viscoelastic properties and mechanical behaviour of resin-rubber blends based on the results of dynamic mechanical analysis (DMA) of melamine resin/phenol-formaldehyde resin/nitrile rubber blend, and to optimise the parameters of the created model using a genetic algorithm.

### Genetic Algorithms

Genetic Algorithms (GAs) are stochastic global search and optimisation techniques that belong to the larger class of Evolutionary Algorithms studied within the field of Evolutionary Soft Computing—a family of computational models inspired by Charles Darwin’s theory of natural evolution and Mendel’s rules of genetics [11]. It is the ability of self-development and adaptation to specific conditions as well as limitations of the solved problem that places GAs among the computational techniques of Artificial Intelligence (AI) [12].

The main component of GAs is the population of chromosomes, or individuals, that represent the individual points of the state space of permissible, correct, and incorrect solutions to the given problem. Each chromosome is represented by an ordered set of parameters that characterise its properties. The elements of this set—genes, or strings, which encode the properties of individual chromosomes—can have a binary, integer, real-number, symbolic, or even combined character, depending on the type of problem to be solved [13].

The initial population of chromosomes in the first computational cycle of GA, or zero-generation of individuals, is usually obtained by random generation of genes within the considered boundaries of the state space of permissible solutions (or search space) [14].

For each solution, which is decoded from the chromosome and inserted into the computer model of the given problem, GA computes the value of the objective function, or fitness function, which determines how well this solution fulfils whatever criteria the algorithm is optimising for, thus quantifying the evolutionary fitness of an individual in adapting to the conditions and limitations of the problem [15].

Depending on the fitness value, the genetic selection operator of GA selects the chromosomes most suitable for reproduction, which either directly or after cross-linking and subsequent mutation pass on their genes to the next generation of the population (next iteration of GA) [16]. Selection of the most suitable chromosomes, identical copies of which will automatically pass to the next generation, or application of a genetic elitism operator, prevents the accidental loss of the best solutions in the random selection [17]. The pseudo-random selection process, simulating natural selection, privileges the best, high-fitness chromosomes, but leaves some chance for reproduction even for low-fitness and worst chromosomes, ensuring a high probability of surviving of the fittest ones and maintaining the high genetic diversity of the population, which is necessary for the implementation of changes in genetic material leading to the minimisation of the risk of the population entrapment in the local optimum. The share of stronger and weaker chromosomes in the population is influenced by the selection pressure, which can significantly increase GA performance and leads to rapid population convergence to a globally optimal solution [18].

A method for sharing information between pseudo-randomly selected chromosomes, called genetic crossover operator, is applied to combine the features of parents to form their offspring, with the possibility that good chromosomes may generate better ones. However, the crossover operator is not usually applied to all selected parent chromosomes. A random choice is made, where the likelihood of crossover being applied depends on probability defined by a design parameter of crossover operator, or crossover rate, which determines if the current parents are combined in the process of their recombination through crossover operator or if they are moved directly to the offspring population. Because good genetic material is found in the mating pool by selection operator, the probability for selecting the fittest parents from the current population is higher than for the worse ones. Accordingly, better solutions are found in every iteration round of GA. Generally, the genetic crossover operator plays a central role in GAs, and its numerous variants have been created to improve GAs behaviour and significantly increase their performance [19].

In order to increase the structural variability of the current population, the genetic mutation operator, which arbitrarily alters one or more genes of randomly selected chromosomes according to a probability defined by a mutation rate parameter, or the mutation probability, is usually used. The mission of mutations in GAs is to replace lost and unused genetic material in the selection and cross-linking of chromosomes in the current population to prevent the premature convergence of GA to suboptimal solutions. As in the case of the crossover operator, there are a number of different variants for mutation, selection, and elitism operators—the choice of a particular variant is determined by the type of problem solved, as well as the way of encoding chromosomes [20].

The process of evaluating the evolutionary success of individuals repeats iteratively until a new generation meets the optimising condition defined by the termination function, or after a predetermined time or a specified number of iterations of GA [21].

At present, very few works can be found that deal with the application of GAs in the field of modelling the mechanical properties of polymeric systems and the prediction of their viscoelastic behaviour under different operating conditions. For example, in the study [22], the GA optimisation method is used for parameter estimation in the modelling of storage modulus of amorphous PPVC and semi-crystalline PP. The GA parameter optimisation procedure has been proposed to determine material parameters of newly modified constitutive models for predicting rate and temperature-dependent stress-strain behaviour of amorphous polymers in the work [23]. The authors of the work [24] apply GA to optimise the viscoelastic parameters, previously obtained using Dynamic Mechanical Analysis measurements, to reliably describe and predict the mechanical behaviour of an energetic polymer material using a general Maxwell model. In the presented work, GA was applied for modelling and prediction of dynamic thermo-mechanical response of resin-rubber blends based on results of Dynamic Mechanical Analysis tests of melamine resin/phenol-formaldehyde resin/nitrile rubber blend modified by high-energy EB radiation for the first time.

## 2. Materials and Methods

### 2.1. Preparation of Samples

Melamine resin, phenol-formaldehyde resin, and nitrile rubber blend, in a ratio of 1:2:2, designated as PMX3 resin-rubber blend, was prepared in the form of granules (Shanghai Jinhu Extrusion Equipment Co., Ltd., Shanghai, China) which were placed into a vulcanisation hydraulic Fontijne Presses, LabEcon Series 600 (Fontijne Presses b.v., Delft, The Netherlands), where it was plasticised for 15 min at a temperature of (383 ± 4) K and a pressure of (80 ± 1) kN. The blend was subsequently homogenised using a laboratory twin roll VOGT LaboWalz W 80 T l (VOGT, Berlin, Germany) into plates of (2 ± 0.04) mm thickness. Prior to radiation treatment, it was allowed to stand for 24 h at a temperature of (298 ± 4) K. From the prepared plates, the rectangular-shaped test samples with dimensions of (30 ± 0.3) mm × (6 ± 0.06) mm × (1.5 ± 0.02) mm for the next DMA analysis were cut using a pneumatic cutter CEAST, Hollow Die Punch-pneumatic 6,054,000 (Instron Ltd., High Wycombe, UK) [25].

### 2.2. Radiation Treatment

The high-energy EB radiation treatment of the PMX3 resin-rubber blend with doses of 77, 138, 150, and 180 kGy was done using a linear electron accelerator UELR-5-1S (NIIEFA, Sankt-Peterburg, Russia) equipped with an indirectly heated Ba-Ni cathode with a diameter of (5 ± 0.1) mm and by a magnetron operating in pulse mode at a frequency of 2998 MHz. The energy of accelerated electrons was (5 ± 0.2) MeV, the pulse duration was (3.5 ± 0.2) μs, and the EB diameter at the outlet through a (50 ± 1) μm thick titanium foil was ≥2 mm. All experiments were done in air at normal pressure and ambient temperature. Radiation doses were calculated by a routine dosimetric system using circles cut out of radiochromic B3 foils (GEX Corporation, Centennial, CO, USA) with a diameter of (1 ± 0.01) cm, which react to radiation by the change of colour. The absorption coefficients of the foils were measured by spectrophoto meter Genesys20 (Thermo Electron Corporation, Madison, WI, USA). The radiation dose was calculated from the experimentally determined dependencies of the dose on absorbance. The combined uncertainty of the applied dosimetric system was less than 6% [25].

### 2.3. DMA Testing

**Figure 1:**
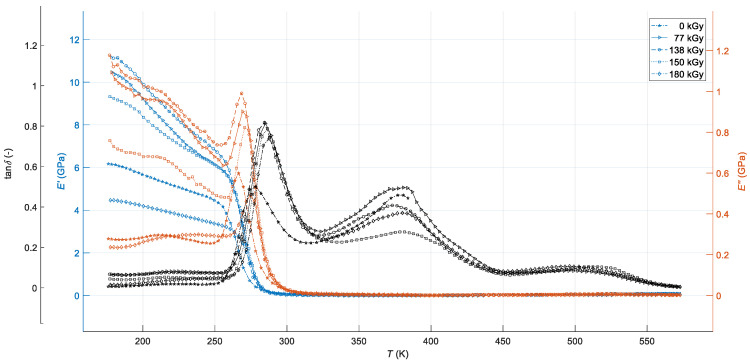
Dynamic mechanical response of PMX3 resin-rubber blend for various EB radiation doses as a function of temperature.

The DMA tests were done using a Q800 Dynamic Mechanical Analyzer (TA Instruments, Baesweiler, Germany). The measurements were carried out in the uniaxial tensile mode, at a constant frequency of dynamic mechanical loading of 1 Hz with an amplitude of 20 μm, in a temperature range from 176 K up to 573 K, and at a constant heating rate of 3 K·min^−1^. The amplitude of applied dynamic stress was 0.1 MPa, and the strain rate was 0.1 s^−1^. The measurements for each radiation dose were performed on 10 specimens. The average values of storage modulus, loss modulus, and loss tangent for each temperature and applied radiation dose were computed; they are presented in Figure 1. The measurement uncertainty was approx. 2%.

## 3. Results and Discussions

### 3.1. Dynamic Mechanical Analysis

The dynamic mechanical response of virgin and high-energy EB-cured samples of the tested PMX3 resin-rubber blend for EB radiation doses of 77, 138, 150, and 180 kGy as a function of temperature in the range from 176 K to 573 K and a constant oscillating strain frequency of 1 Hz is shown in Figure 1.

The DMA curves presented in Figure 1 show features characteristic of amorphous, high-molecular-weight, lightly cross-linked, and multi-phase-separated thermosetting polymer systems [26]. All three *E′*(*T,d*), *E″*(*T,d*) and tan*δ*(*T,d*) dependencies for temperature *T* and each radiation dose *d* show three distinct regions, namely: a glassy region at the temperature range from 176 K to about 275 K with high storage and loss moduli and very low values of loss tangent, where the large-scale segmental mobility of the polymeric chains is highly restricted, such that the tested material in bulk is in a glassy state, and it exhibits a hard, rigid and brittle mechanical behaviour; a glass transition region at temperatures from approximately 266 K to 299 K with a rapid drop in storage modulus by a factor up to 126 at temperature about 292 K toward its initial value and sharp peaks in loss modulus and loss tangent, where the long-range coordinated molecular motion of main chains starts; accordingly, the material occurs in the leathery state and its behaviour changes from the glass-like, hard, rigid and brittle to flexible, rubber-like soft and ductile; and a wide rubbery-plateau region at temperatures above roughly 286 K with a stable, small values of both moduli and non-monotonic changes in loss tangent, where the large-scale chain movements occur, such that the material is in the rubbery state, its stiffness stays very low, and it retains soft and ductile, rubber-like mechanical behaviour [27]. The high level of ductility of the non-irradiated blend in the rubbery-plateau region, described in more detail in our previous work [25], led to automatic interruption of the DMA test at a temperature of approximately 384 K. Moreover, on each of *E″*(*T*,*d*) and tan*δ*(*T*,*d*) curve, two broad shoulders in the glassy region, corresponding to the relatively weak inflexion points on the respective *E′*(*T,d*) curve, can be observed, whereas two broad peaks on tan*δ*(*T,d*) curves in the rubbery-plateau region occur, with the exemption of the non-irradiated sample with a single not-too-wide peak at the end of the curve. The temperature range of the individual transition regions, as well as the height and width of the individual relaxation peaks, obviously show a non-linear dependence on the size of the applied radiation dose, which is due to the different radiation sensitivity of the individual components of the investigated hybrid polymer mixture to the applied EB radiation [6].

The presence of two independent clearly segregated relaxation peaks with different widths and degrees of irregularity in each loss tangent curve in the temperature range of about 250–450 K indicates an inhomogeneous, partially incompatible, and partly immiscible polymeric system with a three-phase-separated morphology [28], which consists of a hard melamine resin phase, a hard phenol-formaldehyde resin phase, and a soft nitrile rubber phase. Generally, the broadening of the DMA damping peaks corresponding to the glass transition events is the indication of the compatibilisation of the multi-component polymeric systems associated with molecular mixing and chemical interactions of its individual components. At the same time, the presence of the two above-mentioned independent peaks with a significant interphase between them in this temperature range of the DMA test indicates that the level of the tested PMX3 blend compatibilisation did not markedly alter the level of its hard and soft phase components’ miscibility, whereas the width and shape of the second of the peaks indicate a relatively high degree of miscibility of both hard phases of the blend, which determines its resulting mechanical properties and behaviour at different temperature conditions [29].

**Figure 2:**
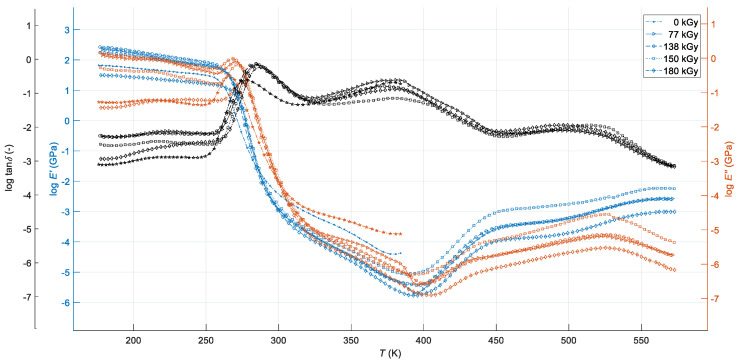
Dynamic mechanical response of PMX3 resin-rubber blend for various radiation doses as a function of temperature in a logarithmic scale.

The relaxation peaks on all DMA curves in the glassy state at temperatures roughly between 176–253 K can be associated with the secondary γ-transition at temperature *T*_γ_, where localised bond movements (bending and stretching) and side-chain movements occur, and with the β-transition at temperature *T*_β_ due to vibrational and rotational motion of water as well as water-polymer blend complex molecules [30], samples of which were not dried before the curing process. The α-transition peaks on *E″*(*T*,*d*), as well as tan*δ*(*T,d*) curves in the leathery state of the blend at temperatures approximately from 266 K to 275 K and from 278 K to 288 K, respectively, correspond to the glass transition of nitrile rubber at temperature *T*_g(NBR)_ [31]. Mostly very indistinctly double, asymmetric damping peaks with faint interphase between them in the rubbery state of the blend at temperatures approximately between 317 K and 455 K can be associated with the glass transition of melamine resin at temperature *T*_g(MFR)_ [32] and phenol-formaldehyde resin at temperature *T*_g(PF)_ [33], respectively. However, these temperatures are, depending on the size of the radiation dose, shifted closer to each other compared to the glass transition temperatures of the individual resins of the blend due to the relatively high degree of miscibility of their molecules and the chemical interactions between their macromolecular segments affected by ionising radiation [8]. At the same time, all three glass transition temperatures identified from the top of peaks on loss tangent curves are significantly shifted towards higher values compared to temperatures identified from peaks on loss modulus curves or from inflexion points on storage modulus curves. The asymmetrical shape of loss tangent peaks in the temperature range between 317 K and 455 K is the result of the inconsistent curing as well as non-stoichiometric blending, which led to the formation of an inhomogeneous polymer network with areas of various cross-link density [34]. The presence of two broad shoulders on loss tangent curves in the rubbery-plateau region of the blend requires a more detailed analysis of functional dependencies *E′*(*T,d*) and *E″*(*T,d*) in its entire temperature range, which is allowed to display the registered DMA curves in the logarithmic scale shown in Figure 2.

As one can be seen from Figure 2, both the storage and loss moduli in the temperature range of approximately 286 K to 395 K and from 286 K to 405 K, respectively, continue to decrease with increasing temperature, except for the non-irradiated sample, whose DMA test was prematurely terminated at a temperature close to 384 K for the above reason. The factor of the maximum decrease of the storage modulus toward its initial value at temperature of approximately 393 K reached a value of almost 3144. Moreover, there is one broad shoulder on each log(*E′*(*T,d*)) and log(*E″*(*T,d*)) curves in the temperature range from about 320 K to 395 K and 320 K to 405 K, respectively, corresponding with the *T*_g(MFR)_ and *T*_g(PF)_ α-relaxation peaks on the log(tan*δ*(*T,d*)) curves, from which it can be inferred that while soft nitrile rubber phase above temperature *T*_g(NBR)_ shows rubbery flow behaviour, the hard melamine and phenol-formaldehyde resin phases can be found in the glass transition state—the presence of rubber thus reduces the overall brittleness of the resin-rubber blend over a wide temperature range. Continued softening of the blend until the temperature close 395 K, the asymmetrical shape of loss tangent peaks at temperatures roughly between 278 K and 288 K, and low storage modulus in the whole temperature range of rubbery flow region, with a maximum value of only about 12 MPa, indicate incomplete curing of all irradiated samples [35]. After reaching its minimum value close to 393 K, the storage modulus increases with increasing temperature as a result of additional, thermally-induced polymerisation reactions. A rather rapid, non-monotonous increase in the *E′*(*T,d*) from this temperature represents the gelation [36] followed by the vitrification [37] of uncured parts of the investigated polymer system, which is documented by two wide double peaks on all log(*E′*(*T,d*)) and log(*E″*(*T,d*)) curves corresponding to the peaks on log(tan*δ*(*T,d*)) curves in the corresponding temperature range of the DMA test. The peak on each log(*E″*(*T,d*)) curve at temperatures around 500 K to 550 K, corresponding to inflexion points at log(*E′*(*T,d*)) curves and the onset of a sharper decline of log(tan*δ*(*T,d*)), can be associated with rubber-(gel)-to-glass transition of the blend, when the rate of the cure reaction significantly decreases due to limited molecular mobility resulting from the additional formation of a solid 3D-network, so that the stiffness of the tested material increases with increasing temperature until reaching a steady value at a plateau temperature close to 550 K at the end of the DMA test [38]. Very low values *E′*(*T,d*) in the final phase of the test, compared to the values of storage modulus in the glassy state, show that additional, thermally-induced polymerisation of the investigated blend at temperatures above about 395 K from a practical point of view almost does not affect its viscoelastic behaviour at a given external dynamic mechanical load (Figure 1). Therefore, this phenomenon, the clarification of which would require further investigation, was not the subject of a more detailed analysis. The issue of modelling of thermally- induced polymerisation is similarly analysed, for example, in [39].

Figure 1 and Figure 2 clearly show that the high-energy EB radiation produces changes in the viscoelastic properties and dynamic mechanical behaviour of the investigated resin-rubber blend, with these changes manifesting themselves in different ways in its various physical states. If we take into account the main topic of the presented work, the variations of viscoelastic properties and dynamic mechanical behaviour with temperature and radiation dose can be effectively characterised by a series of significant points on DMA *E′*(*T,d*) curves enabling their physically-based modelling, which is described in detail in the following paragraph. Its quantitative results are discussed in the text at more appropriate position in Section 3.5, where a detailed analysis of the effect of the size of the radiation dose on the monitored physical quantities of the investigated resin rubber blend is also given.

### 3.2. Stiffness-Temperature Model

We proved in our earlier works [40,41,42] that the temperature dependence of the storage modulus *E′*(*T*) at a given constant oscillating frequency of dynamic mechanical loading for thermoplastic elastomer systems within the whole temperature range of its service life can be quantitatively described with a high level of reliability by the Mahieux and Reifsnider’s unified analytical model [43] based on a Weibull distribution of the failures of secondary bonds between macromolecular chains throughout the primary as well as secondary relaxation processes. The presented study focuses on the ability of such approach to predict the temperature dependence of dynamic mechanical behaviour of much more complex PMX3 resin-rubber polymeric system for the first time, cured by different doses of high-energy EB radiation, which represents, in terms of molecular forces, a specific blend of three different polymers of two different types—rather incompatible and immiscible hybrid blend of one elastomer with two relatively very high compatible and miscible thermosets—while its viscoelastic properties are determined not only by the temperature but also by the size of the applied radiation dose. The changes in storage modulus with temperature *T* and applied radiation dose *d* for such a complex polymeric system can be quantified via Mahieux and Reifsnider’s stiffness-temperature model modified into the form of:

(1) $$E^{\prime }\left(T,d\right)\;=\;\sum_{i\;=\;1}^{N}\Delta E_{i}^{\prime }\left(d\right)\;e^{-\;{\left(\frac{T}{\Theta_{i}\left(d\right)}\right)}^{m_{\;i}\left(d\right)}}$$

where *E′*(*T,d*) is the instantaneous storage modulus at absolute temperature *T* and radiation dose *d*, Δ*E′_I_* is the storage modulus magnitudes of particular transition steps, *Θ_i_* represents absolute transition temperatures, *m_i_* represents the Weibull moduli corresponding to the statistics of the secondary bond breakage, while *N* is the number of observed transition steps. The physical nature of model (1) is based on the fact that in order for the relaxations to occur, the secondary bonds need to break. Therefore, this model is not suitable for the temperature range of additional, thermally-induced polymerisation (Figure 2), when there is an additional formation of new secondary bonds between the macromolecular chains of the investigated blend and further formation of a polymer 3D network increasing its stiffness. In other words, the validity of model (1) is limited by the temperature interval with the predominant thermally-induced degradation processes in the polymeric blend accompanied by a decrease in its stiffness due to the destruction of the secondary bonds with the subsequent disintegration of the polymer network. However, the very small effect of thermally-induced polymerisation on the values of *E′*(*T,d*) at temperatures above 395 K documented above makes it possible to apply the concerned model, in an acceptable approximation, practically in the entire temperature range of the life of the investigated hybrid polymer blend.

Equation (1) can be formally modified into a more practical form:

(2) $$E^{\prime }\left(T,d\right)\;=\;\sum_{i\;=\;1}^{N}\left[E_{i}^{\prime }\left(d\right)\;-\;E_{i\;+\;1}^{\prime }\left(d\right)\right]\;e^{-\;{\left[\frac{T}{\Theta_{i}\left(d\right)}\right]}^{\;m_{\;i}\left(d\right)}}$$

where as

(3) $$E_{N\;+\;1}^{\prime }\left(d\right)\;=\;0$$

In the case of DMA tests of PMX3 resin-rubber blend, performed under the conditions described above, seven relevant transition steps were registered in the temperature range until the beginning of the thermally-induced cross-linking process, so *N* = 7. The significance of other parameters in Equation (2) is as follows: *E′*_1_–*E′*_7_ are the instantaneous storage moduli at the beginning of *E′*(*T,d*) curves, immediately after the γ-transition and β-transition of the blend, immediately after the glass transition of nitrile rubber, at the beginning of the rubbery flow region of the bled and immediately after the glass transition of melamine and phenol-formaldehyde resin, respectively. *Θ*_1_–*Θ*_7_ represent the γ-transition and β-transition temperature of the blend, the glass transition temperature of nitrile rubber, rubbery flow temperature of the blend, glass transition temperature of melamine resin and phenol-formaldehyde resin, and onset temperature of the thermally-induced polymerisation of uncured parts of the blend, respectively; *m*_1_–*m*_7_ are the Weibull moduli corresponding to the statistics of the secondary bonds breaking in the relevant transition regions of the blend.

There are two types of parameters needed to apply the stiffness-temperature model (1) in the form of Equation (2) for describing stiffness changes with temperature and radiation dose, namely: statistical parameters (Weibull moduli) and well-defined physical quantities (instantaneous storage moduli and transition temperatures), which can be identified directly from DMA curves presented in Figure 1 and Figure 2 (physical quantities) or estimated from experimental data (statistical parameters) using any of the optimisation methods, such as a non-linear least-squares fitting technique [40]. However, the standard fitting techniques with commonly used traditional searching algorithms in searching for the optimal solution to problems with a high number of unknown parameters are usually unbearably time-consuming, or get stuck in the local extreme of the optimised function, so they become practically unusable. Therefore, in the presented work, both physical and statistical parameters were estimated in the process of multi-parametric fitting Equation (2) to the experimental *E′*(*T,d*) DMA curves using a global search GA, as an extremely powerful and highly efficient computing method of artificial intelligence suitable for solving complex optimisation problems with a large number of unknown parameters, which may include multi-parametric fitting of non-linear dynamic experimental data produced in DMA testing of polymers.

Generally, the aim of experimental curve fitting techniques is to find the parameters of a model function in such a way that they minimise the total error over the set of experimental data points being considered. Once a model function and an error metric have been selected, the curve fitting becomes an optimisation problem over the given experimental data points. Because GAs are extremely successful as global optimisation techniques with implemented technology of eliminating the risk of entrapment in the local extreme through the application of multiple genetic operators, they are well-suited to curve fitting when it is structured as a problem of estimating unknown coefficients or function parameters of the function-fit model data by searching for the global extreme of a suitably formulated fitness function for such a problem [44].

### 3.3. Curve Fitting by Genetic Algorithm

The estimation of stiffness-temperature model parameters *E′_i_*, *Θ_i_* and *m_i_* in Equation (2) from experimental data sets for each temperature *T* and radiation dose *d* was done by minimising the error between measured, and simulated *E′*(*T,d*) results using a real-coded GA with a fitness function in the form of a sum-squared error:

(4) $$f\left(E_{i}^{\prime },\Theta_{i},m_{i},T,E^{\prime },d\right)\;\;=\;\sum_{j\;=\;1}^{L}{\left\{E_{j}^{\prime }\left(d\right)\;\;-\;\sum_{i\;=\;1}^{N}\left[E_{i}^{\prime }\left(d\right)\;-\;E_{i\;+\;1}^{\prime }\left(d\right)\right]\;e^{-\;{\left[\frac{T}{\Theta_{i}\left(d\right)}\right]}^{\;m_{\;i}\left(d\right)}}\right\}}^{2}$$

where *L* is the *E′*(*T,d*) length or a number of experimental data points. The representation of GA chromosomes with real-valued floating-point numbers was applied because such a representation does not require any data conversion, which in this case significantly shortens the computational time of GA convergence to the optimal solution in the global minimum of fitness function (4), namely only for a few seconds [13].

As mentioned above, the optimisation problem for curve fitting model (1) in the form of Equation (2) to the experimental data sets *E′*(*T,d*) by GA can be mathematically defined as a problem of minimisation of the fitness function (4), that is, for each applied radiation dose *d*, GA solves the problem:

(5) $$\min\left[\;f\left(P,T,E^{\prime }\right)\;\;\in {ℜ}^{n}\right]$$

subject to independent variable values of vector **T**, dependent variable values of vector **E′(T)**, vector **P** of parameters *E′_i_*, *Θ_i_* and *m_i_* of the fitness function (4)

(6) $$P\;=\;\left(E_{i}^{\prime },\Theta_{i},m_{i}\right)\;\;\in {ℜ}^{n}$$

and the vectors of lower bounds

(7) $$LB\;=\;\left(E_{i\min}^{\prime },\Theta_{i\min},m_{i\min}\right)\;\;\in \;\Omega$$

and upper bounds

(8) $$UB\;=\;\left(E_{i\max}^{\prime },\Theta_{i\max},m_{i\max}\right)\;\;\in \;\Omega$$

of intervals of variation each of these 21 parameters of the vector **P**, wherein

(9) LB  ≤ P ≤ UB  ∈ Ω.

The symbol ℜ^n^ represents the set of real numbers, and Ω is the symbol for the space of feasible solutions. Vectors are denoted in bold, regular type as ordered sets of real numbers. The parameters *E′_i_* and *Θ_i_* were bounded by the limits of the intervals **LB** and **UB** of the permissible values from the immediate vicinity of *E′*_1_–*E′*_7_ and *Θ*_1_–*Θ*_7_ for the individual doses *d*_1_–*d*_5_. The limits of these intervals were determined by the method of trial and error so as to ensure a smooth and rapid convergence of GA to the optimal solution or finding the global minimum of the fitness function (4). The values of **LB**(*E′_i_*_min_) and **UB**(*E′_i_*_max_) for *E′*_1_–*E′*_7_ were determined from the individual experimental curves *E′*(*T,d*) on the basis of the corresponding temperatures identified from the respective log(*E″*(*T,d*)) curves. GA testing has shown that the identification of **LB**(*Θ_i_*_min_) and **UB**(*Θ_i_*_max_) for *Θ*_1_–*Θ*_7_ on the basis of experimental log(*E″*(*T,d*)) curves gives significantly better results than their identification from tan*δ*(*T,d*) curves. Each of the *m_i_* parameters was first bounded by a wide interval of positive real numbers with zero ℜ_+,0_, as long as their more precise specification on the basis of experimental data is not possible. Subsequently, the initially estimated **LB**(*m_i_*_min_) and **UB**(*m_i_*_max_) for *m*_1_–*m*_7_ were individually adjusted so that the difference between the experimental and simulated DMA curves *E′*(*T,d*) for each temperature *T* and the radiation dose *d* was minimal.

### 3.4. Optimisation Procedure

The GA starts with a uniformly generated initial population of *PopSize* = 200 real-coded chromosomes (vectors **P**) in the form of double-precision floating-point vectors limited by corresponding **LB** and **UB** vectors values. At the same time, this number of chromosomes also represents a fixed number of individuals (feasible solutions) in each subsequent generation (iteration of the algorithm). Each chromosome in the current population is scored by the fitness function generating its fitness value. Subsequently, the rank scaling function sorts these raw fitness scores in the ascending order and scales them based on the rank, i.e., on the position of each chromosome in the sorted scores list, ordering the chromosomes from the fittest to the least-fit. The rank scaling function assigns scaled values so that the scaled value *s_v_* of a chromosome with rank *r* is proportional to *r^−^*^1/2^, which means that the lowest value fitness maps to rank 1. The sum of the scaled values over the entire population (set of individuals) must equal the number of parents *N_par_* required to create the next generation, which is performed by:

(10) $$s_{v}\left(i\right)\;=\;N_{par}\frac{s_{v}\left(i\right)}{\sum_{i\;=\;1}^{PopSize}s_{v}\left(i\right)}\;$$

Basically, the rank fitness scaling removes the effect of the spread of the raw fitness scores and converts them into a more usable range of scaled or expectation values that is suitable for the selection operator’s function [45].

Each new generation of chromosomes *G_τ_*_+1_ is the result of applying a set *ξ* of three main types of genetic operators—selection operator *S*, crossover operator *C*, and mutation operator *M*—to the previous generation *G_τ_*, which can be formally presented as a functional dependence of:

(11) $$G_{\tau +1}\;=\;g\left(G_{\tau },\xi \right)$$

for

(12) ξ ∈ (S,C,M) 

where *τ* is the serial number of the generation.

A stochastic uniform selection function was used to choose parents for the next generation based on their scaled values from the rank scaling function because it ensures that fit parent chromosomes always get picked at least once. That ensures that each chromosome has as much chance to be selected as a parent as any other and guarantees that both high fitness and low fitness individuals will be part of the selection for each generation with sufficiently high diversity. The stochastic uniform selection function computes a line in which each parent corresponds to a section of the line of length proportional to its expectation value. The GA moves along that line in equal size steps, one step for each parent, and the algorithm allocates a parent to each step from the section it lands on. The first step is a uniform random number R less than the step size StepSize determined by dividing the total of expectation values into the number of parents *N_par_* needed to create the next generation, or [46]:

(13) $$R\;<\;StepSize\;\mathrm{for}\;StepSize\;=\;\frac{\sum_{i\;=\;1}^{PopSize}s_{v}\left(i\right)}{N_{par}}$$

An elite count *p_child_* = 0.05∙*PopSize* and a crossover fraction *p_cross_* = 0.8 specifies the number of chromosomes with the best expectation values that are guaranteed to survive to the next generation as elite children, and the fraction of the next generation, other than elite children, that crossover operator produces, respectively. The remaining individuals in the next generation are produced by mutation operator.

A constraint-dependent crossover function creates a random binary vector, then selects the genes (individual elements of vectors **P**) where this vector is a 1 from the first parent and the genes where the vector is a 0 from the second parent; subsequently, with the probability equal to crossover fraction *p_cross_*, the crossover function combines the selected genes to form the first child, and vice-versa to form the second one [47].

A constraint-dependent adaptive feasible mutation function randomly generates directions that are adapted with respect to the last generation. The feasible region of each variable of the vector **P** is bounded by corresponding **LB** and **UB** vectors values. A step length is chosen along each direction so that the **LB** and **UB** bounds are satisfied. This mutation process with an adaptive mutation rate consists of a random generation of a mutation direction vector and an initial step size. Then a mutated individual is generated and if it is located in the infeasible region, the step size is adjusted to a smaller value and generates another mutated individual along the mutation direction vector. The previous step is repeated until the generated individual is within the feasible region [48].

The GA runs until the average relative change in the fitness function value over stall generation (the generation for which there is no improvement in the best fitness value) is less or equal than fitness function tolerance of 10^−6^ [49].

The GA optimisation procedure described above was performed in the “Genetic Algorithm—Optimisation Toolbox” of the software package Matlab^®^ Version 7.10.0.499 R2010a 64-bit (MathWorks, Natick, MA, USA), which provides a complete set of tools for sufficiently efficient work with GAs of a wide range of configurations, including problems of multi-parametric fitting the experimental curves. The Matlab^®^ software was chosen primarily because of its exceptionally high global penetration in the academic, development, and industrial environments, as well as for minimal demands on the user’s programming skills. Settings of selected GA parameters in Matlab^®^ Optimisation Toolbox is clear from the above text, while the rest of the GA parameters are the default ones.

### 3.5. Curve Fitting Results

**Figure 3:**
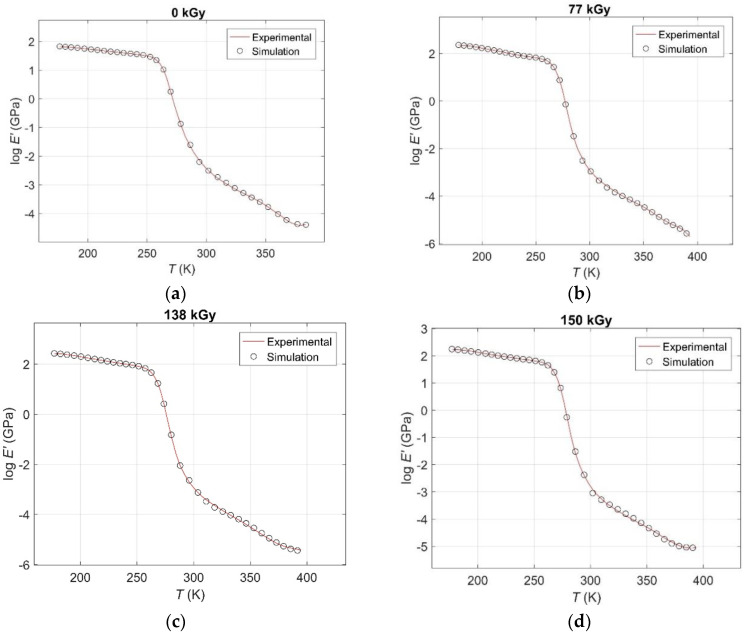

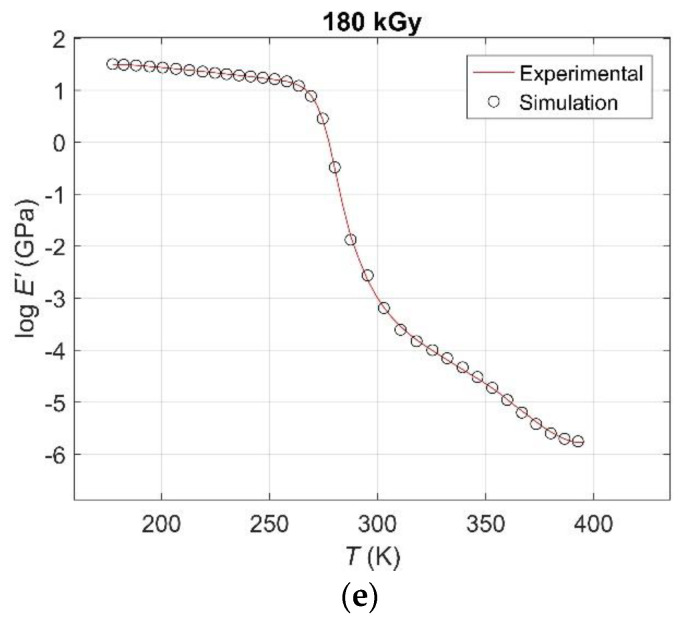
The results of curve fitting by GA compared to the corresponding experimental log(*E′*(*T,d*)) curves in the temperature range until the onset temperature of the additional thermally-induced polymerisation for: (**a**) an unirradiated sample; (**b**) for a radiation dose of 77 kGy; (**c**) for a radiation dose of 138 kGy; (**d**) for a radiation dose of 150 kGy; (**e**) for a radiation dose of 180 kGy.

**Figure 4:**
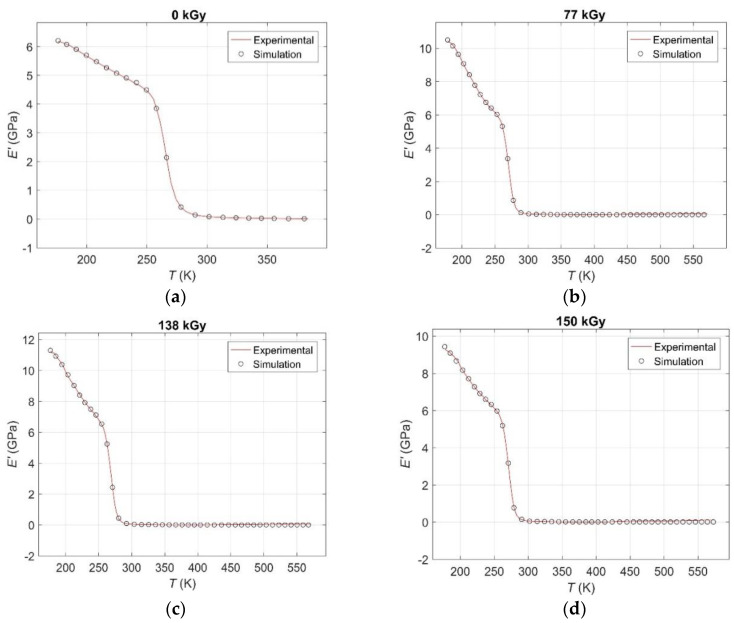

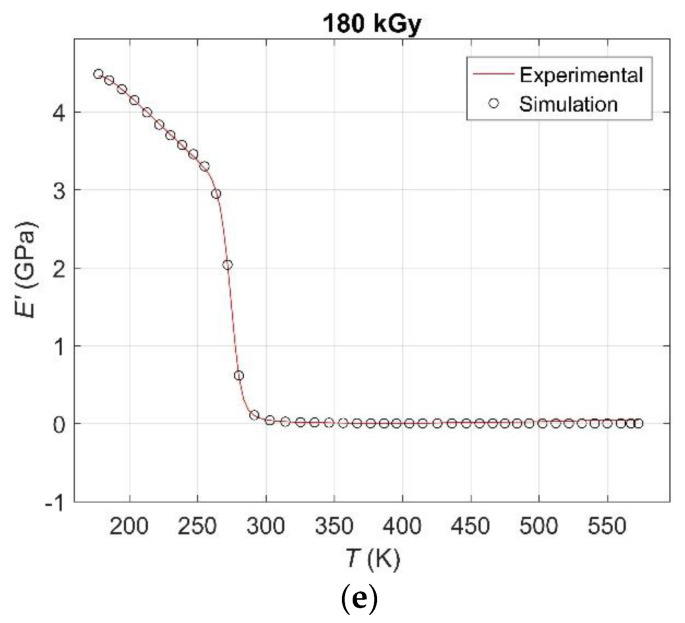
The results of curve fitting by GA compared to the corresponding experimental ones *E′*(*T,d*) curves over the entire investigated temperature range for: (**a**) an unirradiated sample; (**b**) for a radiation dose of 77 kGy; (**c**) for a radiation dose of 138 kGy; (**d**) for a radiation dose of 150 kGy; (**e**) for a radiation dose of 180 kGy.

The results of curve fitting by global search GA compared to the corresponding experimental ones *E′*(*T,d*) curves are given in Figure 3 and Figure 4, from which an excellent match between real and simulated data is evident.

**Figure 5:**
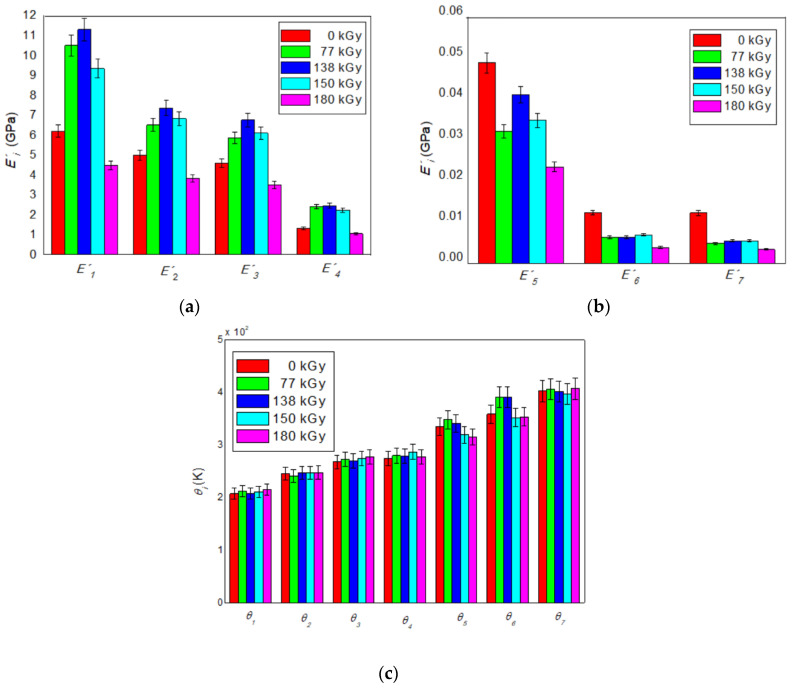
The influence of the radiation dose size on values of instantaneous storage moduli: (**a**) E′1–E′4; (**b**) E′5–E′7; (**c**) the influence of the radiation dose size on values of transition temperatures *θ1*–*θ7*.

**Figure 6:**
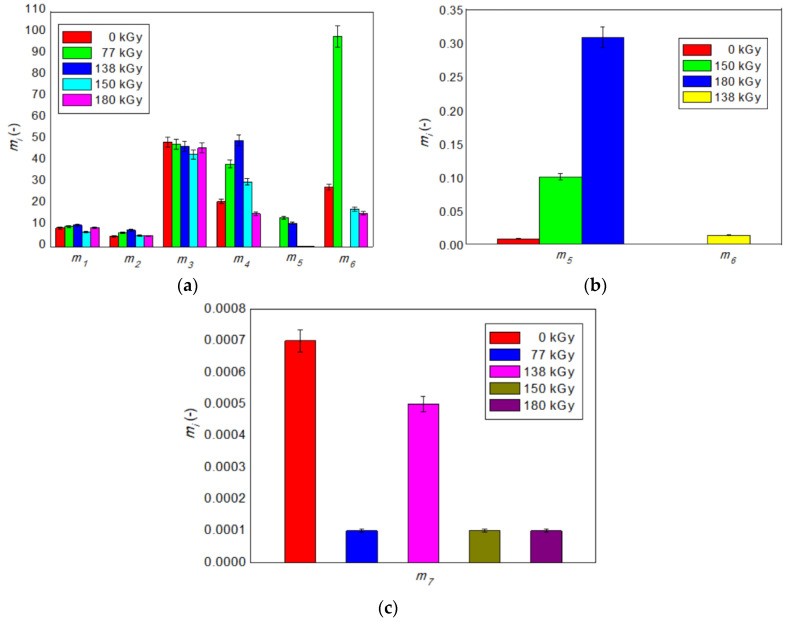
Influence of the radiation dose size on values of Weibull moduli: (**a**) *m*_1_–*m*_7_ at radiation doses of 0, 77, 138, 150 and 180 kGy; (**b**) *m*_5_–*m*_6_ at radiation doses of 0, 138, 150 and 180 kGy; (**c**) Weibull modulus *m*_7_ at radiation doses of 0, 138, 150 and 180 kGy.

The influence of the radiation dose on the monitored physical properties of the investigated resin-rubber PMX3 blend, as well as on the values of statistical parameters of the distribution of the breaking of secondary bonds is presented in Figure 5 and Figure 6, respectively.

Figure 5 clearly shows that the values of instantaneous storage moduli *E′*_1_–*E′*_4_ until a radiation dose of 138 kGy grows non-linearly, with *E′*_1_ being higher by almost 83%, *E′*_2_ by 48%, *E′*_3_ by 47%, and *E′*_4_ by up to 89% than the values of the non-irradiated blend. Thus, radiation doses up to 138 kGy significantly increase stiffness as in the glassy and rubbery region of the blend, as well as in the glass transition region of its nitrile rubber phase due to the prevailing radiation-induced cross-linking reactions accompanied by the creation of a polymer 3D network. From a radiation dose above 138 kGy, the values of all four storage moduli mentioned above show a non-linear decreasing trend. Values of storage moduli *E′*_5_–*E′*_7_ decrease non-monotonically with increasing radiation dose, while the highest decrease, up to almost 73% compared to the value of the non-irradiated blend, shows a modulus *E′*_7_ at a radiation dose of 180 kGy. Thus, at the same time, irradiation of the investigated polymer blend with high-energy EB radiation significantly increases the ductility in its rubbery flow region and reduces stiffness in both the glass transition region of its melamine phase and phenol-formaldehyde phase. Decrease of moduli *E′*_1_–*E′*_7_ is a consequence of the prevailing radiation-induced degradation processes, resulting in the simultaneous decomposition of the polymer 3D network that is being formed.

Changes in values of transition temperatures *θ*_1_–*θ*_4_ and *θ*_7_ with the level of the radiation dose oscillate in intervals of errors of their estimation at the level of less than 5% compared to the values of the non-irradiated blend. An increase in value *θ*_5_ at radiation doses of 77 and 138 kGy does not reach the magnitude of the error, while its decrease at higher radiation doses is less than 6%, so *θ*_5_ shows a very slightly declining trend. In contrast, the values *θ*_6_ at radiation doses of 77 and 138 kGy increase by almost 10% with a consequent decrease practically to the level of the non-irradiated blend, so that after the radiation dose of 138 kGy, they show an increasing trend. Irradiation of the investigated polymer blend with high-energy EB radiation thus significantly affects only the glass transition temperature of phenol-formaldehyde resin phase, which shifts towards higher values at radiation doses up to 138 kGy with a subsequent decrease at higher radiation doses and, at the same time, slightly reduces the values of the glass transition temperature of melamine resin phase.

Estimated values of Weibull moduli *m*_1_ and *m*_2_, corresponding to the statistics of the secondary bonds breaking during the γ- and β-transition, are relatively low as expected: the strength of the bonds that need to be broken to allow rearrangement of side groups for the secondary relaxations depends on the relative position of each side group to the other molecular chains; therefore, the number of segments involved in the γ- and β-transition is very low, and distribution of the strength of the bonds in the glassy state of the blend is wide with low values of both *m*_1_ and *m*_2_ moduli. In order to be capable of a long-range, coordinated segmental motion during the glass transition, and to be able to reptate or diffuse in the rubbery region, the molecules also need to break intermolecular bonds as in the case of the secondary relaxations, but over a large part of each molecule. Therefore, the number of segments involved in the glass transition of nitrile rubber, as well as in motions in the rubbery state of the blend, is much higher; the distribution of the strength of the bonds is much narrower, so values of Weibull moduli *m*_3_ and *m*_4_ are substantially larger. Moduli *m*_5_ and *m*_6_, corresponding to the statistics of the secondary bonds breaking during the glass transition of melamine and phenol-formaldehyde resin, depending on the size of the radiation dose, vary from an extremely low to an extremely high value due to the different degree of cross-linking of the two hard phases of the blend. In the rubbery flow state of the blend, virtually all secondary bonds have already disintegrated, so that the assemblies of chains can move in a coordinated manner, with their free movement being hindered only by the polymer network formed; therefore, the intensity of secondary bond failures is close to zero, and the distribution of the bond strength is very broad with almost zero modulus value *m*_7_ [50].

It is also clear from Figure 5 and Figure 6 that, with the exception of *m*_3_ and *m*_6_, the trend of change in the values of the Weibull modulus with the increasing value of the applied radiation dose corresponds to the trend of change of storage moduli *E′_i_* in individual transition regions of the blend. The detected mismatch for moduli *m*_3_ a *m*_6_ may be due to different parameter identification methodologies of *E′_i_* and *m_i_*—namely, instantaneous storage moduli *E_i_* are determined by extreme values *E′_i_*_min_, *E′_i_*_max_ of intervals of feasible solutions **LB** and **UB**, which were searched on log(*E″*(*T,d*)) curves, while Weibull moduli *m_i_* were estimated directly from *E′*(*T,d*) curves by genetic curve-fitting method, so the estimated and actual values may partly differ from each other. However, based on the above analysis, it can be concluded that the Weibull moduli *m_i_*, as purely statistical parameters, reflect the observed physical processes taking place at different temperatures and applied radiation doses in the investigated polymer blend at the microstructural level relatively very well.

## 4. Conclusions

In the presented work, the effects of high-energy electron beam radiation on the viscoelastic properties and dynamic mechanical behaviour of resin-rubber blends were studied. The variations of the storage modulus, loss modulus, and loss tangent with temperature and radiation dose—obtained from the dynamic mechanical analysis of the melamine resin, phenol-formaldehyde resin, and nitrile rubber blend over the entire temperature spectrum, including the transitions, at a single constant frequency of its dynamic mechanical loading, strain rate, and heating rate—were analysed for the first time. A new stiffness-temperature model based on Weibull statistics of the secondary bonds breaking during the relaxation transitions has been developed to quantitatively describe changes in the storage modulus as a function of temperature and radiation dose until the onset temperature of the additional, thermally-induced polymerisation of uncured parts of the blend. The real-coded global search genetic algorithm has been successfully used to estimate the unknown parameters of the developed model by the multi-parametric genetic curve-fitting method. The excellent agreement between the modelled and experimental data has been found over the entire investigated temperature range. It was also found that the high-energy electron beam radiation with doses to 138 kGy allows targeted modification of the dynamic mechanical properties of the investigated resin-rubber blend with a significantly predominant effect of formation of the radiation-induced polymeric network and that the effect of the additional, thermally-induced polymerisation to their resulting values is practically negligible. A significant radiation-induced increase in the stiffness of the blend at lower temperatures with a simultaneous slight increase in its ductility at high temperatures can play an important role, e.g., in extending the service life of friction composite systems based on resin-rubber matrices used mainly in the automotive industry. However, the physical nature of the created stiffness-temperature model makes it possible to assume that in conjunction with the global search genetic algorithm in the process of optimising its parameters, it can find practical application in the study of a wider range of hybrid polymeric blends modified by high-energy ionising radiation.
